# Differential expression of plasma miR-146a in sepsis patients compared with non-sepsis-SIRS patients

**DOI:** 10.3892/etm.2013.937

**Published:** 2013-01-30

**Authors:** LINA WANG, HUA-CHENG WANG, CHA CHEN, JIANMING ZENG, QIAN WANG, LEI ZHENG, HUAN-DU YU

**Affiliations:** 1 Department of Laboratory Medicine, Guangdong Provincial Hospital of Traditional Chinese Medicine, Guangzhou 510006;; 2 Department of Laboratory Medicine, Nanfang Hospital, Southern Medical University, Guangzhou 510515;; 3 Department of Laboratory Medicine, Guangzhou Brain Hospital, Guangzhou 510370;; 4 Department of Laboratory Medicine, Kingmed Diagnostics, Guangzhou 510006, P.R. China

**Keywords:** miR-146a, sepsis, diagnosis

## Abstract

Sepsis is a subtype of systemic inflammatory response syndrome (SIRS), which is characterized by infection. Circulating microRNAs (miRNAs), including miR-150, miR-146a and miR-223, are potential biomarkers of sepsis. In this study, we demonstrated that measuring the relative expression of miR-146a/U6 in plasma, using the 2^−ΔΔCt^ method, provides a method for differentiating between sepsis and non-sepsis-SIRS. We observed a significant increase in miR-146a expression in the initial cohort of 6 non-sepsis-SIRS patients compared to the 4 sepsis patients (P=0.01) and in the second cohort of 8 non-sepsis-SIRS patients compared to the 10 sepsis patients (P=0.027). Additionally, we identified that sodium citrate and ethylenediaminetetraacetic acid (EDTA) K_2_ may be used as anticoagulant reagents. Generation of a standard curve is not necessary in these diagnostic tests, unless the standard of normalization is carefully selected. Thus we provide more detailed guidance for the clinical use of circulating miRNA biomarkers.

## Introduction

Sepsis is a life-threatening condition and the major cause of mortality among patients in intensive care units (ICUs). The gold standard to distinguish sepsis from non-infectious diseases is blood microbiological culture analysis, which is more time-consuming than other potential biomarkers for early detection of sepsis, including acute phase proteins such as C-reactive protein (CRP) and procalcitonin (PCT). However, the lack of specifity of these more frequently used biomarkers impedes the significant requirement to identify novel biomarkers for early sepsis detection.

microRNAs (miRNAs) are a class of small (21–23 nucleotides), non-protein-coding RNAs that regulate the expression of target mRNAs post-transcriptionally. miRNAs are associated with a number of biological processes and the expression signatures of miRNAs serve as diagnostic and prognostic markers for various diseases. Numerous studies have established that the levels of miRNAs in serum or plasma are stable (4–7). After being normalized or quantified, circulating miRNAs yield high sensitivity and specificity by microarrays, next-generation sequencing or quantitative reverse transcription-polymerase chain reaction (RT-PCR) methods. miR-146a is one of the miRNAs that regulates the inflammatory response through a negative feedback nuclear factor (NF)-κB pathway (8), by directly downregulating interleukin-1 receptor-associated kinase 1 (IRAK1), tumor necrosis factor receptor-associated factor 6 (TRAF6) (8,9) and IRAK2 (9). miR-146a may serve as a biomarker for rheumatoid arthritis (RA) (10) and sepsis (11). To investigate the clinical implication of miR-146a as a biomarker for sepsis diagnosis, we developed a qPCR-based precise quantification assay that reliably quantifies miR-146a (normalized to U6) from archived patient samples. We identified that plasma/serum miR-146a levels from sepsis patients were slightly decreased compared with non-sepsis-SIRS samples. However, different set points should be determined according to the control samples used during each experiment in clinical diagnostic practice.

## Materials and methods

### Participants

Blood samples were collected from 28 patients from Guangzhou Brain Hospital and Guangdong Provincial Hospital of Traditional Medicine, China. All patients provided informed consent. The study was approved by the ethics committee of Guangdong Provincial Hospital of Traditional Medicine, Guangzhou, China. Plasma samples in cohort 1 were collected and archived in April 2011 and plasma samples for cohort 2 were collected and archived between May and June 2011. All samples were collected when the first blood microbiological culture samples were collected. The results of blood microbiological culture determined whether patients had sepsis or non-sepsis-SIRS.

### Plasma collection and RNA isolation

Both sodium citrate and ethylenediaminetetraacetic acid (EDTA) K_2_ anticoagulant samples were collected from the same patient at a particular time. Blood samples were centrifuged at 1,760 × g for 10 min at room temperature. Plasma were obtained and mixed with TRIzol reagent (Dongsheng Biotech Co., Ltd., Guangzhou, China) at a ratio of 1:2 and stored at −80°C. Total RNA was extracted from serum according to the manufacturer’s instructions.

### Real-time quantitative RT-PCR

The RNA extracted from plasma were reverse transcribed as follows: a mixture consisting of 2 *μ*l RNA, 2 *μ*l stem-loop RT primer (RiboBio Co., Ltd., Guangzhou, China) and 15 *μ*l ddH_2_O were mixed and incubated at 70°C for 10 min and then maintained at 4°C. Then, an additional 16.5 *μ*l ddH_2_O, 10 *μ*l 5X RT buffer, 2 *μ*l 2.5 nM deoxynucleotide triphosphate (dNTP) and 2.5 *μ*l 200 U/*μ*l PrimeScript RTase (Takara Bio Inc., Japan) were added, for a total volume of 50 *μ*l. RT was performed on a Veriti^®^ Thermal Cycler (Applied Biosystems, Carlsbad, CA, USA) at 30°C for 10 min, 42°C for 60 min, 70°C for 15 min and then held at 4°C.

The EvaGreen PCR Master Mix (Bio-Rad, Hercules, CA, USA) was used to analyze the expression of miRNA. The U6 and miR-146a specific stem-loop amplification primers (RiboBio Co., Ltd.) were diluted to the working concentration. Then 9 *μ*l EvaGreen Master Mix was mixed with 4 *μ*l miRNA specific primers, 2 *μ*l RT product and 5 *μ*l ddH_2_O in RNase-free strip-tubes. The qRT-PCR was then performed at 95°C for 20 sec, at 95°C for 10 sec and at 60°C for 34 sec, with the last two steps repeated for 40 cycles. Then, the dissociation curve was generated. Data were analyzed with SDS software (Applied Biosystems), using the automatic Ct setting for assigning the threshold for Ct determination.

Standard curves were generated using synthetic miR-146a (RiboBio Co., Ltd), serially diluted and input into the RT reaction mixtures at the volume of 2 *μ*l per reaction.

### Statistical analysis

Data were analyzed using the Mann-Whitney U test for the comparison of the different groups. The Wilcoxon matched pairs test was used to compare differences between the different anticoagulant groups. SPSS 13.0 software (SPSS Inc., Chicago, IL, USA) was used for all statistical analyses. Relative quantification using the 2^−ΔΔCt^ method in the sepsis vs. non-sepsis-SIRS groups was carried out and fold changes were calculated for miR-146a.

## Results

### Normalization of miR-146a qRT-PCR data

Raw Ct values of U6 small nuclear RNA (snRNA) demonstrated no significant differences between the sepsis and non-sepsis-SIRS groups (P=0.067 in cohort 1 and P=0.122 in cohort 2; Kruskal-Wallis test). Paired samples were collected in sodium citrate or ethylenediaminetetraacetic acid (EDTA) K_2_ anticoagulant tubes, respectively, from the same patient. No significant differences were detected in raw Ct values of U6 snRNA or miR-146a between the two anticoagulant groups.

**Figure 1:**
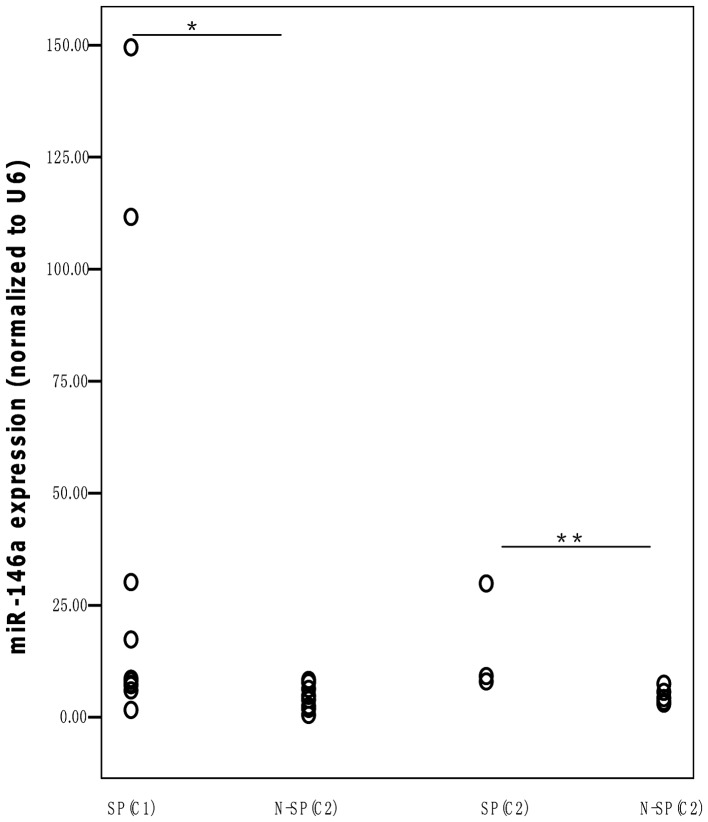
Comparison of miR-146a expression measures by real-time qRT-PCR. Data were normalized to U6 and were presented as 2^[Ct(miR - 146a) - Ct(U6)]^. The levels of miR-146a were significantly reduced in the sepsis plasma samples. Triplicate assays were performed on each RNA sample. SP (C1), sepsis plasma samples in cohort 1 (n=4); SP (C2), sepsis plasma samples in cohort 2 (n=6); N-SP (C1), non-sepsis-SIRS plasma samples in cohort 1 (n=10); N-SP (C2), non-sepsis-SIRS plasma samples in cohort 2 (n=8). Significant increases in miR-146a expression are indicated by ^*^P=0027 and ^**^P= 0.01. qRT-PCR, quantitative reverse transcription-polymerase chain reaction; SIRS, systemic inflammatory response syndrome.

In cohort 1, plasma miR-146a, normalized to U6 snRNA, demonstrated significant differences between the two groups, when normalized values were calculated as 2^[Ct(miR-146a) - Ct(U6)]^. Additionally, in cohort 2, plasma miR-146a in the sepsis group was significantly lower compared to that in the non-sepsis-SIRS group, when the same normalized values were used (Fig. 1).

### Absolute quantification of miRNAs

**Figure 2:**
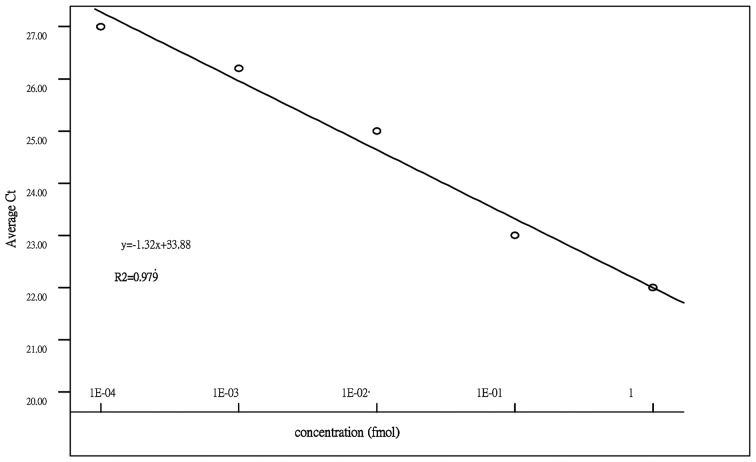
Standard curve for qRT-PCR assays generated for miR-146a using a dilution series of miR-146a mimics purchased from RiboBio Co., Ltd. qRT-PCR, quantitative reverse transcription-polymerase chain reaction.

The absolute expression levels of miR-146a were also calculated according to the standard curve of miR-146a (Fig. 2). No significant differences between the sepsis and non-sepsis-SIRS patients were identified in either cohort (P=0.762 in cohort 1 and P=0.696 in cohort 2). Additionally, no significant difference was observed when the absolute miR-146a levels were normalized to total RNA (P=0.067 in cohort 1 and P=0.274 in cohort 2).

### Diagnostic value of miR-146a for sepsis

**Figure 3:**
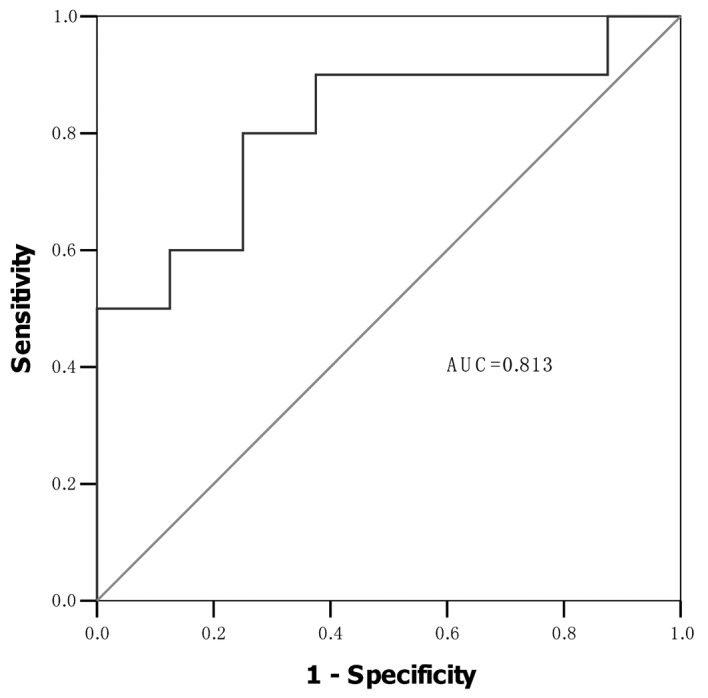
Receiver operating characteristics (ROC) curve analysis using serum miR-146a for discriminating sepsis patients from non-sepsis-SIRS patients. The AUC (area under the ROC curve) was 0.813 (95% CI, 0.608-1.017) with a specificity of 87.5% and sensitivity of 60%, respectively. SIRS, systemic inflammatory response syndrome.

Receiver operating characteristics (ROC) curve analysis indicated that the area under the curve (AUC) of miR-146a was 0.813 and at a cut-off point set at 7.97, miR-146a yielded a specificity of 87.5% and sensitivity of 60%, respectively (cohort 2; Fig. 3).

## Discussion

SIRS occurs when the body experiences an ongoing inflammatory response, which may arise from infectious or non-infectious triggers, including pancreatitis, trauma, drug fever and immunologic reactions. Sepsis is a potentially fatal condition characterized by a whole-body inflammatory state (12–14).

Sepsis may lead to multiple organ dysfunction syndrome (MODS) and mortality. A previous study demonstrated that for every hour delay in the appropriate antibiotic treatment of sepsis there is a 7% rise in mortality (15). The risk of mortality as a result of sepsis is strongly affected by the underlying disease. The 30-day survival rates for severe sepsis and septic shock patients are 65–80 and 40–60%, respectively. The case-fatality rates are similar for culture-negative and culture-positive severe sepsis (16).

The majority of Gram-negative bacteria produce endotoxin, which causes fever and shock that are common symptoms during severe infections. Endotoxin binds to CD14 and then interacts with toll-like receptor 4 (TLR4). TLR2 is involved in the systemic response to Gram-positive bacteria infections (17). Studies are currently being performed to elucidate the mechanism of the response to these organisms.

Several circulating miRNAs, including miR-150, miR-182, miR-342-5p and miR-486 in peripheral blood mononuclear cells (PBMCs), miR-150 in plasma (18), and miR-146a and miR-223 in serum (11) are associated with sepsis. Nahid *et al* (19) identified that the miR-146a level was continuously elevated in THP-1 cells until 24 h after lipopolysaccharide (LPS) treatment. Taganov *et al* (8) identified that miR-146a/b, miR-132 and miR-155 are endotoxin-responsive genes and miR-146 is involved in the signaling cascade of TLR4/MyD88/IRAK1/TRAF6, which triggers the activation of IκB kinase, c-jun N-terminal kinases (JNK), NF-κB and activator protein (AP)-1, resulting in an upregulation of immune-responsive genes. Through a NF-κB/IRAK1/TRAF6/ miR-146a negative feedback regulation loop, the activity of the inflammatory response is reduced.

There are several types of calibrators for absolute quantification of miRNAs by qRT-PCR, including plasmid (20), RNA fraction (21), cDNA samples (22) and synthetic target miRNA (23). For relative qRT-PCR, an appropriate reference gene must be selected. U6 snRNA was selected for the reference gene in this study since no difference in U6 snRNA levels has been detected in the serum/plasma of candidates with certain diseases and in control conditions (24–26), which was confirmed by our data. It was demonstrated that plasma miR-146a/U6 with the 2^−ΔΔCt^ method yielded a similar AUC to serum miR-146a/cel-miR-39 with the 2^−ΔΔCt^ method (11).

The role of miR-146a in the physiopathology of sepsis continues to be poorly understood. However, we demonstrated, using various sample types and normalized methods, that miR-146a may be used as a potential marker to difference sepsis from non-sepsis-SIRS. It may be used in clinical practice as a complementary test for sepsis in patients where a diagnosis is not clear.
